# Trinucleotide cassettes increase diversity of T7 phage-displayed peptide library

**DOI:** 10.1186/1472-6750-7-65

**Published:** 2007-10-05

**Authors:** Lauren RH Krumpe, Kathryn M Schumacher, James B McMahon, Lee Makowski, Toshiyuki Mori

**Affiliations:** 1 Molecular Targets Development Program, Center for Cancer Research, NCI-Frederick, Frederick, Maryland, 21702, USA; 2 SAIC-Frederick, Inc., NCI-Frederick, Frederick, Maryland 21702, USA; 3 Werner H. Kirsten Student Internship Program, NCI-Frederick, Frederick, Maryland, 21702, USA; 4 Biosciences Division, Argonne National Laboratory, Argonne, Illinois, 60439, USA

## Abstract

**Background:**

Amino acid sequence diversity is introduced into a phage-displayed peptide library by randomizing library oligonucleotide DNA. We recently evaluated the diversity of peptide libraries displayed on T7 lytic phage and M13 filamentous phage and showed that T7 phage can display a more diverse amino acid sequence repertoire due to differing processes of viral morphogenesis.

**Methods:**

In this study, we evaluated and compared the diversity of a 12-mer T7 phage-displayed peptide library randomized using codon-corrected trinucleotide cassettes with a T7 and an M13 12-mer phage-displayed peptide library constructed using the degenerate codon randomization method.

**Results:**

We herein demonstrate that the combination of trinucleotide cassette amino acid codon randomization and T7 phage display construction methods resulted in a significant enhancement to the functional diversity of a 12-mer peptide library. This novel library exhibited superior amino acid uniformity and order-of-magnitude increases in amino acid sequence diversity as compared to degenerate codon randomized peptide libraries. Comparative analyses of the biophysical characteristics of the 12-mer peptide libraries revealed the trinucleotide cassette-randomized library to be a unique resource.

**Conclusion:**

The combination of T7 phage display and trinucleotide cassette randomization resulted in a novel resource for the potential isolation of binding peptides for new and previously studied molecular targets.

## Background

Bacteriophage (phage)-displayed random peptide libraries have become a widely-used screening resource for identifying ligands for molecular targets. Several applications of phage-displayed peptide library technology include epitope and protein-protein interaction mapping, and ligand and enzyme substrate specificity identification [1-5]. Successful application of the technology is dependent on screening methodology, on the biophysical characteristics of the target molecule and peptide library, and on the amino acid sequence diversity of the library. The library should be of adequate sequence diversity to contain binding ligands for the target, however, diversity can be limited both by biological censorship of phage-displayed amino acid sequences and by methods of amino acid sequence randomization.

Biological censorship of libraries displayed on phage is a consequence of conducting combinatorial chemistry within a biological host system. Phages displaying amino acid sequences that are detrimental to viral stability or to the processes of viral replication or morphogenesis tend to be lost or suppressed within a library, whereas amino acid sequences that do not interfere with these processes, or those that are advantageous, are preferentially retained and propagated within the library [6,7]. Recently novel statistical methods have been developed to quantitate the amino acid sequence diversity of combinatorial libraries [7-9]. These methods provide estimates of library functional rather than technical diversity, and therefore can describe the behavior of peptide populations in which peptide copy number can vary. Briefly, a library with greater numbers of amino acid sequences having the same probability of occurrence is functionally more diverse than a library with lesser numbers of equally probable sequences. Using these novel statistical methods, we recently demonstrated that the functional diversity of peptide libraries displayed on T7 lytic phage can surpass that of peptide libraries displayed on M13 filamentous phage due to the differing processes of viral morphogenesis [10]. For proof-of-principle, we screened several T7-displayed libraries for streptavidin-binding peptides and isolated previously reported binding motifs as well as novel binding motifs, several of which contained amino acid residues suppressed within M13-displayed libraries [10]. In addition, we have successfully isolated binding epitopes for human lactoferrin from a hexameric random peptide library displayed on T7 phage [11].

Diversity is typically introduced into a phage-displayed peptide library by randomizing library oligonucleotide DNA through the use of a reduced genetic code. For example, the use of degenerate codon NNK (where N represents a 25% mix each of adenine, thymine, guanine, and cytosine nucleotides; and K represents a 50% mix each of thymine and guanine nucleotides) in library DNA construction reduces the standard genetic code from 64 to 32 codons, encodes each of the 20 amino acids, and eliminates 2 of 3 stop codons [12]. Use of such genetic codes is beneficial in that the reduction in amino acid codon number grants each amino acid a more uniform chance of incorporation into the peptide library, and that the reduction in stop codon number minimizes the occurrence of truncated peptides within the library. Thus, NNK randomization can produce superior amino acid sequence diversity as compared to the standard genetic code randomization (Table 1, compare *in silico *libraries).

**Table 1:** Amino acid sequence diversity of proteomes and libraries

	Average diversity per position	Diversity per 12-mer	Functional diversity (# of equally probable sequences)
Proteomes:			
Human	0.85	0.14	5.73 × 10^14^
*E. coli*	0.82	0.09	3.69 × 10^14^
			
Phage-displayed libraries:			
T7 Trinuc	0.91	0.32	1.31 × 10^15^
T7 NNK	0.85	0.14	5.73 × 10^14^
M13 NNK	0.68	0.01	4.10 × 10^13^
			
*In silico *libraries:			
20 codon (trinucleotide)	1.00	1.00	4.10 × 10^15^
32 codon (reduced)	0.83	0.11	4.51 × 10^14^
61 codon (standard)	0.79	0.06	2.46 × 10^14^

Populations of random 12-mer peptides from the human and *E. coli *proteomes and from computationally-random *in silico *libraries were collected as previously described and were subjected to diversity analysis by the DIVAA program of the RELIC web server [4]. DIVAA-generated positional diversity estimates were utilized to calculate the average positional diversity estimates for the peptide populations. Diversity per 12-mer was determined by raising the average positional diversity to the twelth-power. Functional diversity [6] was estimated by multiplying the diversity per 12-mer by the total number of possible sequences (20^12 ^for a 12-mer peptide population). Portions of this table were reproduced with kind permission from Wiley-VCH Verlag GmbH & Co. KGaA, see acknowledgements section for details.

A completely random, and thus maximally diverse, 12-mer peptide library could contain 4.1 × 10^15 ^(20 amino acids ^12 positions^) equally abundant and unique amino acid sequences. This maximum diversity would be obtained if each amino acid position is populated by equal proportions of each amino acid. Previous diversity estimates on a computationally-random *in silico *12-mer peptide library, constructed by selecting codons at random from the 32-codon NNK-randomized reduced genetic code, gave a functional diversity approximately equivalent to 0.11 (11%) or 4.51 × 10^14 ^equally probable peptides [8] (Table 1). Thus, based on bias imparted solely through unequal numbers of codons for different amino acids, the diversity of an NNK-randomized peptide library is decreased by approximately 90%. Codon-corrected trinucleotide cassettes offer an attractive alternative to oligonucleotide randomization. Each amino acid codon is first synthesized as an individual trinucleotide phosphoramidite cassette in accordance with *E. coli *codon preference [13,14]. The cassettes are then mixed at pre-defined ratios, and the mixture is subsequently applied to synthesize the library oligonucleotide DNA codon by codon. Trinucleotide cassette randomization can decrease potential biological censorship of amino acid sequences by avoiding rarely used codons within the host organism. In addition, trinucleotide cassette randomization offers the ability to specify amino acid distribution at each amino acid position and the choice to eliminate undesirable amino acids and stop codons. Because each amino acid codon can be granted an equal chance of incorporation into the library DNA, each amino acid position could be populated by a uniform distribution of amino acids. Trinucleotide cassette randomization should therefore point toward the maximum functional diversity of a phage-displayed peptide library [6]. We herein demonstrate that the combination of T7 phage and the trinucleotide cassette randomization method can result in significant enhancements to the diversity of phage-displayed peptide libraries.

## Results and discussion

**Figure 1:**
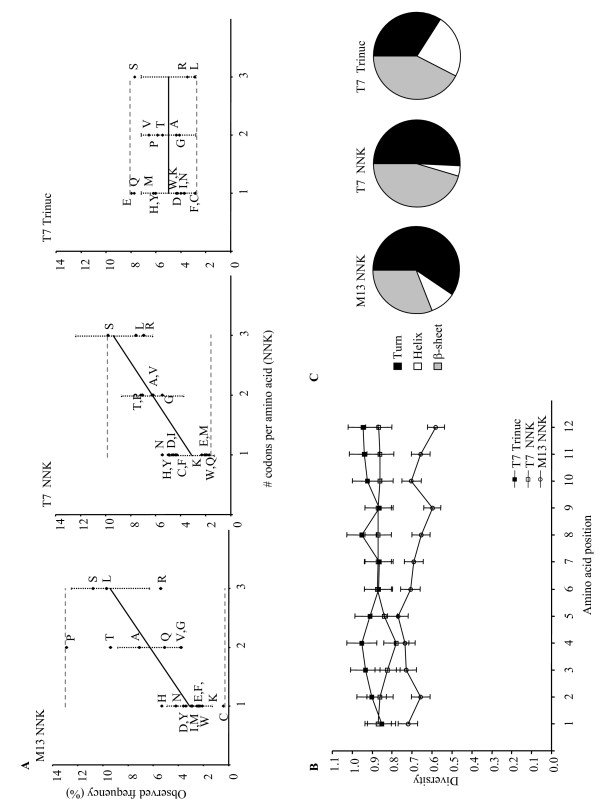
Diversity analyses of the M13 NNK, T7 NNK, and T7 Trinuc random 12-mer peptide library populations. (A) AAFREQ analysis. Vertical dashed lines are error bars which represent the standard deviation of expected amino acid frequencies (Poisson statistics). Horizontal dashed lines represent the range of observed amino acid frequencies for each library peptide population. (B) DIVAA analysis. Error bars indicate the standard deviation in the estimation of diversity [7]. (C) Peptide secondary structure composition predictions. Portions of this figure were reproduced with kind permission from Wiley-VCH Verlag GmbH & Co. KGaA, see acknowledgements section for details.

We collected a series of amino acid sequences from three phage-displayed 12-mer peptide libraries; a commercially-available NNK-randomized M13 phage-displayed library (M13 NNK), an NNK-randomized T7 phage-displayed library (T7 NNK), and the trinucleotide cassette-randomized T7 phage-displayed library (T7 Trinuc). These sequences were submitted for analysis by several bioinformatics programs. First, overall and position-specific amino acid frequencies were tabulated for each peptide population using the Amino Acid Frequency (AAFREQ) program of the RELIC web server. The overall observed amino acid frequencies (amino acid count/total number of amino acids in population) were plotted against the number of codons per amino acid using the NNK-randomized reduced genetic code (Figure 1A). Although the T7 Trinuc library was designed to allow one codon per amino acid, the amino acid frequencies were plotted in the same format as the NNK libraries (1, 2, or 3 codons per amino acid) for ease of comparison. The observed amino acid frequencies should adhere to the expected frequencies dictated by codon number (solid black line) if the population is not subjected to biological censorship. Vertical dashed lines are error bars which indicate the standard deviation in the expected amino acid frequencies per codon number assuming Poisson statistics (square root of expected frequency). The horizontal dashed lines indicate the range of observed amino acid frequencies for each library peptide population.

The expected range of amino acid frequencies for the NNK-randomized libraries was approximately 3.1 ± 1.8% (1 codon/amino acid) to 9.4 ± 3.1% (3 codons/amino acid), assuming Poisson statistics. Within the M13 NNK library peptide population, proline, threonine, and histidine residues were over-represented, whereas arginine and cysteine residues were under-represented. The amino acid frequencies derived from the M13 NNK population ranged from 0.4% (cysteine) to 12.9% (proline). Within the T7 NNK population asparagine and tyrosine residues were over-represented. However, M13-associated amino acid biases were relieved within the T7 NNK population, and the range of amino acid frequencies adhered more strictly to the expected frequencies (1.7% to 9.8%). The expected range of amino acid frequencies for the T7 Trinuc library was 5 ± 2.2% (Poisson statistics). Within the T7 Trinuc peptide population, glutamic acid, glutamine, and serine residues were over-represented, which suggested weak preference for these amino acids. Given that each amino acid was granted equal opportunity for incorporation into the library, the preferences may possibly be attributed to a requirement for stable T7 viral capsid formation, or to tRNA isoacceptor abundance. However, within the T7 Trinuc population, M13-associated amino acid biases were also relieved. These data suggested that both the T7 NNK and T7 Trinuc libraries were potentially subjected to a lesser degree of biological censorship than the M13 NNK library. Of the three libraries analyzed, the T7 Trinuc population demonstrated the most uniform amino acid frequencies, with a range of 2.8% to 8.0%. Increased amino acid frequency uniformity should result in increased functional diversity, and therefore the diversity of the T7 Trinuc library should surpass the diversity of the T7 NNK library.

Next, the AAFREQ outputs for each peptide population were analyzed for position-specific amino acid biases. Any positional amino acid frequency which exceeded the standard deviation of the expected frequency was identified as a potential bias. Within the M13 NNK peptide population (Additional file 1), alanine and tyrosine residues were over-represented, and proline residues were under-represented at position one (amino-terminus) of the peptides. Histidine, asparagine, and threonine residues were over-represented, while glycine, arginine, valine, and tryptophan residues were under-represented within the amino-terminus. Glutamic acid and glutamine were under-represented within the carboxyl-terminus of the peptides. Proline was over-represented at positions two through twelve, and cysteine residues were suppressed at all amino acid positions. These specific amino acid biases can be attributed to censorship of amino acid sequences during the processes of filamentous phage morphogenesis, inner membrane translocation and signal peptide cleavage of the recombinant phage proteins, and *E. coli *infection [6]. Several potential position-specific amino acid biases were observed within the T7 NNK population, but no obvious pattern of bias was identified (Additional file 2). The position-specific amino acid biases observed within the M13 NNK population were absent in the T7 NNK population, which indicated the possibility that peptide display on T7 may increase library sequence diversity [10]. Within the T7 Trinuc population glutamic acid, glutamine, and serine were over-represented at most amino acid positions (Additional file 3). Tyrosine residues appeared over-represented within the amino terminus of the peptides, however, no obvious pattern of position-specific censorship was observed. As with the T7 NNK population, M13-associated positional biases were also relieved within the T7 Trinuc population. One interesting observation was that within both T7-displayed populations, as compared to the M13 NNK population, the number of potential positional amino acid over-representations was increased relative to under-representations. As this analysis only identifies overall or positional amino acid biases, presumably there are populations of censored amino acid sequences within each library that are not biased in any way we could detect.

Each peptide population was then submitted to the RELIC server for diversity analysis by the program Analysis of Amino Acid Diversity in Multiple Aligned Protein Sequences (DIVAA). DIVAA provides an intuitive functional diversity estimate for the distribution of amino acids at each position within the peptide populations [17]. If the position is populated by one amino acid (complete conservation, 1/20), then the diversity estimate is 0.05. If the position is populated by equal proportions of each amino acid (complete randomization, 20/20), then the diversity estimate is 1.0. The estimation of diversity as a function of position allows for the identification of any site-specific censorship of displayed amino acids. Figure 1B shows the DIVAA outputs for each peptide population. Both T7-displayed populations exceeded the diversity of the M13 NNK population at each amino acid position, indicating that each population incorporated a more uniform distribution of amino acids. The T7 Trinuc population exceeded the diversity of T7 NNK population at eight amino acid positions and was essentially equal at four amino acid positions. The positional diversity averages and overall diversity estimates for proteomes, phage-displayed peptide libraries, and *in silico*-generated peptide libraries are given in Table 1. The T7 Trinuc population gave the greatest functional diversity approximately equivalent to 10^15 ^equally probable sequences. In addition, it surpassed the diversity of both NNK-randomized phage-displayed peptide library populations by orders of magnitude (10^13^–10^14 ^sequences), as well as the human and *E. coli *proteome-derived populations and computationally-random *in silico *peptide populations (10^14 ^sequences).

Functional diversity estimates reflect only the degree of uniformity in the distribution of amino acids within a population [17]. As such, we evaluated several biophysical properties of the phage-displayed peptide library populations: predicted secondary structure composition, peptide net charge distribution, and peptide hydropathy distribution (M13 NNK library properties were presented in Krumpe et al., 2006 [10]). Secondary structures were predicted by submitting the amino acid sequences for analysis by the Chou-Fasman approach [16] of the GCG Wisconsin package program PeptideStructure. The output for each peptide was collected and regions of the peptides demonstrating strong propensity for secondary structure formation (denoted by a capital letters in the PeptideStructure output, data not shown) were tabulated for the analysis. Figure 1C shows that both the M13 and T7 NNK-randomized library populations exhibited the greatest propensity for β-turn, followed by β-sheet, and lastly, for α-helix formation. Within the T7 Trinuc population, the propensity to form β-sheets was the greatest, followed by β-turns, and then α-helices, and the percentage of the three secondary structures was more uniform than in the NNK-randomized libraries.

**Figure 2:**
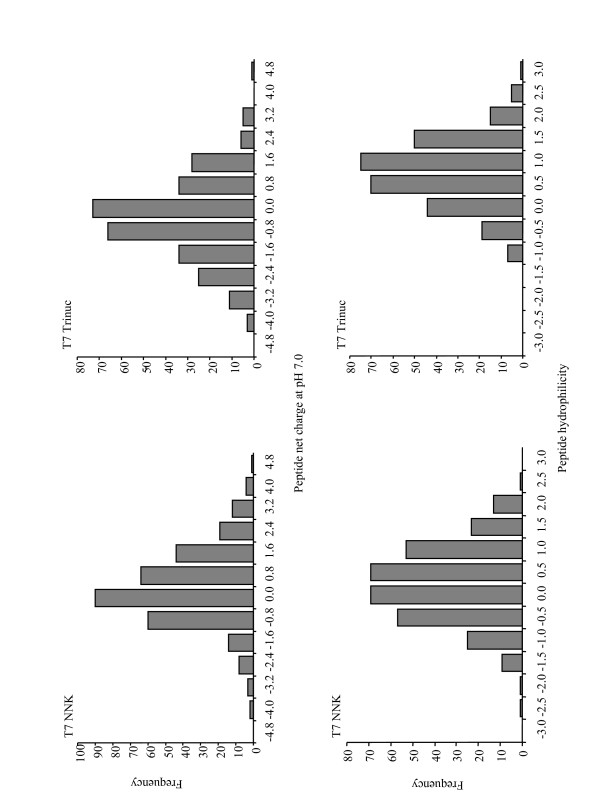
Net charge and hydrophilicity distributions for the T7 NNK and T7 Trinuc library peptide populations. Portions of this figure were reproduced with kind permission from Wiley-VCH Verlag GmbH & Co. KGaA, see acknowledgements section for details.

Peptide net charge and hydrophilicity distributions are shown in Figure 2. An NNK-randomized peptide library was expected to exhibit an average net charge of approximately 0.5 per 12-mer peptide at pH 7.0, based on amino acid net charge and codon frequencies. The T7 NNK library population net charge distribution centered at approximately 0.4 and demonstrated positive skew, which indicated higher frequencies of peptides with net positive charges than peptides with negative net charges (Figure 2, top left). The trinucleotide cassette randomized libraries were expected to exhibit an average net charge of approximately -0.2. The T7 Trinucleotide library population net charge distribution centered at approximately -0.4 and demonstrated negative skew, which indicated higher frequencies of peptides with negative net charges (Figure 2, top right).

The NNK-randomized population of peptide hydrophilicities was expected to produce an average peptide hydrophilicity of approximately 0.3 based on Kyte and Doolittle [15] assigned amino acid hydrophilicities (positive values indicate hydrophilicity and negative values indicate hydrophobicity) and amino acid codon frequencies. The T7 NNK population centered at 0.3 and exhibited a normal distribution of peptide hydrophilicities (Figure 2, bottom left). Trinucleotide cassette randomized populations are expected to give an average peptide hydrophilicity of approximately 0.5 for a more hydrophilic library than an NNK-randomized library. The T7 Trinuc population hydrophilicity distribution centered at approximately 0.8 and exhibited a relatively normal distribution of peptide hydrophilicities. Taken together the data indicated that the T7 NNK library population exhibited net charge and hydrophilicity character that adhered well to the expected library properties. The T7 Trinuc library population showed slightly more peptide negative net charge and hydrophilic character than expected, which is likely the result of the over-abundance of glutamic acid, glutamine, and serine residues within the peptide population. Despite the differences in biochemical properties between the T7 NNK and Trinuc libraries, we isolated an essentially identical set of streptavidin-binding peptides from each library (Additional file 4) [10]. It is undetermined how overall differences in library biophysical properties such as secondary structure, hydropathicity, and net charge will affect phage-displayed peptide library screenings; however it is likely the effect would be target-specific. For example, some targets may preferentially bind hydrophilic ligands and thus a more hydrophilic library may offer a higher chance of success, whereas some targets may prefer hydrophobic ligands and thus a more hydrophobic library such as an NNK-randomized library would be indicated. Regardless, the T7 Trinuc library represents a potential resource of binding peptides with unique biophysical properties.

## Conclusion

All applications of phage-displayed peptide library technology may potentially benefit from the novel method of library construction using the T7 phage display system in combination with codon-corrected trinucleotide cassette randomization. As each amino acid is granted an equal opportunity for incorporation into the phage library DNA, the trinucleotide cassette randomization method allows for more uniform amino acid frequencies as compared to degenerate codon randomization methods. As such, the functional diversity of the T7 Trinuc library peptide population exceeded the functional diversity of the T7 NNK library by 10^14 ^amino acid sequences and exceeded the functional diversity of the M13 NNK library by 10^15 ^amino acid sequences. In addition, the diversity of the T7 Trinuc library was superior to the diversity of human and *E. coli *proteomes. The T7 Trinuc library also may provide a novel resource of peptidic ligands of negative net charges, increased hydrophilicity, and a more even distribution of secondary structures. As previous phage-displayed peptide library screenings may have been influenced by amino acid biases imparted by degenerate codon randomization methods, in addition to biases associated with the M13 filamentous phage life cycle, the use of T7 phage-displayed peptide libraries constructed using codon-corrected trinucleotide cassette randomization offers a resource of novel binding peptides for new and previously studied molecular targets.

## Methods

We constructed a T7 phage-displayed 12-mer peptide library randomized using codon-corrected trinucleotide cassettes (T7 Trinuc) with identical methods as previously described [9]. Briefly, to construct the library oligonucleotide DNA, each amino acid codon position was allowed to incorporate an equal proportion of each of 20 trinucleotide cassettes (Glen Research, Sterling, Virginia). The T7 Select 10-3b cloning system (Novagen, Madison, Wisconsin) was utilized to prepare the library, and 1.2 × 10^9 ^individual recombinants were generated prior to amplification. The library was amplified once in *E. coli *and then plated at low density to randomly select phage clones for DNA sequence analysis. The progeny phages were predicted to display on average 5-15 copies of the random peptides (T7 Select System Manual, TB178, Novagen). A population of 286 deduced amino acid sequences was collected from the T7 Trinuc library and was subjected to bioinformatics-assisted diversity analyses. The REceptor LIgand Contacts (RELIC) web server [18] was used to tabulate amino acid frequencies and to estimate functional diversity. The Genetics Computer Group (GCG) Wisconsin Package programs Isoelectric and PeptideStructure (version 10.3, Accelrys, Inc., USA) were used to calculate peptide net charge at pH 7.0 (sum of positively charged residues minus the sum of negatively charged residues) and to calculate hydrophilicity according to the algorithm of Kyte and Doolittle [15] with the window of integration set to twelve (length of peptides). The Chou-Fasman approach was used to predict peptide secondary structure [16]. The diversity analyses were compared to those of two previously constructed NNK-randomized phage-displayed 12-mer peptide libraries; the T7 library (T7 NNK, 321 peptides) [9] and New England Biolabs' M13 pIII-displayed Ph.D.-12™ library (M13 NNK, 441 peptides, available for download from the RELIC web server [18]).

## Abbreviations

NNK, degenerate amino acid codon; RELIC, REceptor LIgand Contacts; GCG, Genetics Computer Group; AAFREQ, amino acid frequency program; DIVAA, diversity in multiple aligned sequences program.

## Authors' contributions

LK provided statistical analyses of the data and prepared the manuscript. KS constructed and sequenced the T7 Trinuc library and prepared a draft manuscript. JM approved the research. LM offered oversight and helpful discussion. TM designed and coordinated the research. All authors read and approved the final manuscript.

## Supplementary Material

Additional file 1 Observed positional amino acid frequencies for the 441-member M13 NNK library peptide population. This table gives positional amino acid frequencies as well as the expected positional frequency and standard deviation. Click here for file

Additional file 2 Observed positional amino acid frequencies for the 321-member T7 NNK library peptide population. This table gives positional amino acid frequencies as well as the expected positional frequency and standard deviation. Click here for file

Additional file 3 Observed positional amino acid frequencies for the 286-member T7 Trinuc library peptide population. This table gives positional amino acid frequencies as well as the expected positional frequency and standard deviation. Click here for file

Additional file 4 Streptavidin-binding peptides isolated from the T7 Trinuc library. This table gives the amino acid sequence, binding frequency, and ELISA binding values for streptavidin-binding peptides isolated from the T7 Trinuc library. Click here for file

## References

[B1] Szardenings M (2003). Phage display of random peptide libraries: applications, limits, and potential. J Recept Signal Transduct Res.

[B2] Krumpe LRH, Mori T (2006). The Use of Phage-Displayed Peptide Libraries to Develop Tumor Targeting Drugs. Int J Peptide Res Ther.

[B3] Ladner RC, Sato AK, Gorzelany J, de Souza M (2004). Phage display-derived peptides as therapeutic alternatives to antibodies. Drug Discov Today.

[B4] Mori T (2004). Cancer-specific ligands identified from screening of peptide-display libraries. Curr Pharm Des.

[B5] Krumpe LRH, Mori T (2007). Potential of peptide library technology to identify functional targeting peptides. Exp Opin Drug Discovery.

[B6] Rodi DJ, Soares AS, Makowski L (2002). Quantitative assessment of peptide sequence diversity in M13 combinatorial peptide phage display libraries. J Mol Biol.

[B7] Rodi DJ, Makowski L (1999). Phage-display technology–finding a needle in a vast molecular haystack. Curr Opin Biotechnol.

[B8] Makowski L, Soares A (2003). Estimating the diversity of peptide populations from limited sequence data. Bioinformatics.

[B9] Mandava S, Makowski L, Devarapalli S, Uzubell J, Rodi DJ (2004). RELIC–a bioinformatics server for combinatorial peptide analysis and identification of protein-ligand interaction sites. Proteomics.

[B10] Krumpe LRH, Atkinson AJ, Smythers GW, Kandel A, Schumacher KM, McMahon JB, Makowski L, Mori T (2006). T7 Lytic Phage-Displayed Peptide Libraries Exhibit Less Sequence Bias than M13 Filamentous Phage-Displayed Peptide Libraries. Proteomics.

[B11] Sakamoto K, Ito Y, Mori T, Sugimura K (2006). Interaction of human lactoferrin with cell adhesion molecules through RGD motif elucidated by lactoferrin-binding epitopes. J Biol Chem.

[B12] Barbas CF, Burton DR, Scott JK, Silverman GJ (2001). Appendix 1, in Phage display: a laboratory manual.

[B13] Kayushin AL, Korosteleva MD, Miroshnikov AI, Kosch W, Zubov D, Piel N (1996). A convenient approach to the synthesis of trinucleotide phosphoramidites–synthons for the generation of oligonucleotide/peptide libraries. Nucleic Acids Res.

[B14] Virnekas B, Ge L, Pluckthun A, Schneider KC, Wellnhofer G, Moroney SE (1994). Trinucleotide phosphoramidites: ideal reagents for the synthesis of mixed oligonucleotides for random mutagenesis. Nucleic Acids Res.

[B15] Kyte J, Doolittle RF (1982). A simple method for displaying the hydropathic character of a protein. J Mol Biol.

[B16] Chou PY, Fasman GD (1978). Prediction of the secondary structure of proteins from their amino acid sequence. Adv Enzymol Relat Areas Mol Biol.

[B17] Rodi DJ, Mandava S, Makowski L (2004). DIVAA: analysis of amino acid diversity in multiple aligned protein sequences. Bioinformatics.

[B18] REceptor LIgand Contacts (RELIC) web server. http://relic.bio.anl.gov/.

