# Supplementary material for: Trinucleotide cassettes increase diversity of T7 phage-displayed peptide library
Source: BMC Biotechnol. 2007 Oct 5;7:65. doi: 10.1186/1472-6750-7-65 (PMC2174457; doi:10.1186/1472-6750-7-65)
Supplement: Additional file 1 — Observed positional amino acid frequencies for the 441-member M13 NNK library peptide population. This table gives positional amino acid frequencies as well as the expected positional frequency and standard deviation. [file 1472-6750-7-65-S1.pdf]

Additional Table 1. Observed positional amino acid frequencies for the 441-member M13 NNK library peptide population. Position 1 corresponds to the amino-terminus of the peptides. Expected positional amino acid frequencies were calculated based on amino acid codon frequency in the NNK-randomized reduced 32-codon genetic code and on the total number of peptides analyzed (amino acid codon frequency x number of peptides analyzed). Ranges for expected amino acid frequencies were calculated assuming Poisson statistics (square root of expected frequency %). Over-represented amino acids are highlighted in red, and under-represented amino acids are highlighted in blue. Portions of this table were reproduced with kind permission from Wiley-VCH Verlag GmbH & Co. KGaA, see acknowledgements section for details.

AA	#1	#2	#3	#4	#5	#6	#7	#8	#9	#10	#11	#12	Expected
A	45	27	31	31	20	23	31	37	37	29	30	32	28 ± 11
C	3	1	2	1	1	0	5	0	2	3	1	1	14 ± 8
D	18	17	19	19	24	17	8	8	7	23	12	9	14 ± 8
E	19	16	17	11	14	11	6	10	3	4	8	3	14 ± 8
F	11	8	14	7	7	15	6	15	14	13	9	7	14 ± 8
G	25	15	5	15	14	12	16	17	19	22	23	19	28 ± 11
H	25	21	23	32	25	34	27	15	20	19	16	22	14 ± 8
I	18	22	18	13	15	14	13	10	8	13	11	6	14 ± 8
K	6	14	10	18	17	19	7	6	4	10	14	6	14 ± 8
L	40	38	36	44	32	43	46	44	53	46	39	52	41 ± 14
M	11	14	20	13	11	12	18	15	6	12	15	8	14 ± 8
N	34	19	21	27	21	25	20	14	9	8	11	11	14 ± 8
P	1	71	48	49	55	52	60	64	74	61	74	76	28 ± 11
Q	26	17	22	20	34	21	21	24	27	17	27	13	28 ± 11
R	10	17	15	17	24	15	30	31	31	25	33	35	41 ± 14
S	55	51	55	47	43	42	35	57	40	48	42	54	41 ± 14
T	42	45	46	41	39	54	50	32	41	38	38	33	28 ± 11
V	16	12	22	10	21	9	20	14	19	20	19	21	28 ± 11
W	11	4	3	5	9	4	10	15	7	17	7	21	14 ± 8
Y	25	12	14	21	15	19	12	13	20	13	12	12	14 ± 8
