# Supplementary material for: Trinucleotide cassettes increase diversity of T7 phage-displayed peptide library
Source: BMC Biotechnol. 2007 Oct 5;7:65. doi: 10.1186/1472-6750-7-65 (PMC2174457; doi:10.1186/1472-6750-7-65)
Supplement: Additional file 2 — Observed positional amino acid frequencies for the 321-member T7 NNK library peptide population. This table gives positional amino acid frequencies as well as the expected positional frequency and standard deviation. [file 1472-6750-7-65-S2.pdf]

Additional Table 2. Observed positional amino acid frequencies for the 321-member T7 NNK library peptide population. Position 1 corresponds to the amino-terminus of the peptides. Expected positional amino acid frequencies were calculated based on amino acid codon frequency in the NNK-randomized reduced 32-codon genetic code and on the total number of peptides analyzed (amino acid codon frequency x number of peptides analyzed). Ranges for expected amino acid frequencies were calculated assuming Poisson statistics (square root of expected frequency %). Over-represented amino acids are highlighted in red, and under-represented amino acids are highlighted in blue. Portions of this table were reproduced with kind permission from Wiley-VCH Verlag GmbH & Co. KGaA, see acknowledgements section for details.

AA	#1	#2	#3	#4	#5	#6	#7	#8	#9	#10	#11	#12	Expected
A	20	20	21	15	20	19	26	23	19	16	21	22	20 ± 8
C	18	7	14	14	15	9	13	14	18	15	11	22	10 ± 6
D	15	21	7	15	19	13	15	16	11	14	20	15	10 ± 6
E	8	6	5	4	10	9	5	5	7	7	6	7	10 ± 6
F	19	13	19	12	11	10	12	11	14	19	12	14	10 ± 6
G	25	16	14	19	14	18	19	19	19	19	15	14	20 ± 8
H	17	21	18	16	15	14	16	20	12	9	16	16	10 ± 6
I	10	9	15	16	17	17	16	14	15	21	17	9	10 ± 6
K	5	9	8	8	5	12	7	6	8	3	8	9	10 ± 6
L	31	23	29	28	18	28	22	21	22	24	20	25	30 ± 10
M	6	6	5	5	4	9	7	8	8	6	11	5	10 ± 6
N	15	19	16	16	15	19	20	15	17	22	15	22	10 ± 6
P	22	21	21	24	30	21	25	24	29	20	16	18	20 ± 8
Q	9	5	2	6	9	6	4	4	7	11	7	4	10 ± 6
R	24	15	20	21	21	21	27	21	22	26	28	22	30 ± 10
S	27	25	31	45	33	32	28	31	31	32	35	27	30 ± 10
T	16	22	32	22	25	28	17	22	21	22	27	23	20 ± 8
V	17	33	16	18	26	19	15	18	20	17	15	28	20 ± 8
W	5	9	8	3	3	7	7	6	2	4	4	8	10 ± 6
Y	12	21	20	14	11	10	20	23	19	14	17	11	10 ± 6
