# Supplementary material for: Trinucleotide cassettes increase diversity of T7 phage-displayed peptide library
Source: BMC Biotechnol. 2007 Oct 5;7:65. doi: 10.1186/1472-6750-7-65 (PMC2174457; doi:10.1186/1472-6750-7-65)
Supplement: Additional file 3 — Observed positional amino acid frequencies for the 286-member T7 Trinuc library peptide population. This table gives positional amino acid frequencies as well as the expected positional frequency and standard deviation. [file 1472-6750-7-65-S3.pdf]

Additional Table 3. Observed positional amino acid frequencies for the 286-member T7 Trinuc library peptide population. Position 1 corresponds to the amino-terminus of the peptides. Expected positional amino acid frequencies were calculated based on amino acid codon frequency, which was 5% (1/20) for each amino acid using trinucleotide cassette randomization, and on the total number of peptides analyzed (5% x 286). Ranges for expected amino acid frequencies were calculated assuming Poisson statistics (square root of expected frequency %). Over-represented amino acids are highlighted in red, and under-represented amino acids are highlighted in blue.

AA	#1	#2	#3	#4	#5	#6	#7	#8	#9	#10	#11	#12	Expected
A	10	13	10	14	11	12	16	17	8	14	11	14	14 ± 6
C	7	9	3	7	13	9	10	9	5	6	9	8	14 ± 6
D	9	13	14	16	13	8	11	10	6	13	11	13	14 ± 6
E	17	26	21	16	27	28	23	21	24	24	25	21	14 ± 6
F	11	6	11	7	8	8	5	8	5	8	10	11	14 ± 6
G	13	9	15	12	5	11	11	12	16	18	9	10	14 ± 6
H	10	18	16	22	14	17	24	13	14	24	21	14	14 ± 6
I	10	8	10	11	10	11	9	15	10	12	10	11	14 ± 6
K	5	9	11	10	10	19	10	11	11	15	16	19	14 ± 6
L	7	7	13	10	8	4	6	5	12	12	8	7	14 ± 6
M	30	14	17	17	24	16	15	16	14	18	17	14	14 ± 6
N	8	13	11	10	10	9	11	15	4	13	15	9	14 ± 6
P	12	19	19	15	17	28	20	17	21	12	10	11	14 ± 6
Q	22	20	23	23	22	25	27	23	25	21	17	18	14 ± 6
R	9	9	7	13	11	10	8	11	14	6	11	11	14 ± 6
S	26	27	22	12	16	16	26	19	26	23	26	25	14 ± 6
T	18	15	13	15	21	11	10	17	20	9	17	21	14 ± 6
V	21	22	16	12	15	21	22	22	21	14	17	22	14 ± 6
W	17	15	11	19	20	10	9	8	13	8	10	10	14 ± 6
Y	24	14	23	25	11	13	13	17	17	16	16	17	14 ± 6
