# Supplementary material for: Trinucleotide cassettes increase diversity of T7 phage-displayed peptide library
Source: BMC Biotechnol. 2007 Oct 5;7:65. doi: 10.1186/1472-6750-7-65 (PMC2174457; doi:10.1186/1472-6750-7-65)
Supplement: Additional file 4 — Streptavidin-binding peptides isolated from the T7 Trinuc library. This table gives the amino acid sequence, binding frequency, and ELISA binding values for streptavidin-binding peptides isolated from the T7 Trinuc library. [file 1472-6750-7-65-S4.pdf]

Additional Table 4: Streptavidin-binding peptides isolated from the T7 Trinuc library

Sequence	Binding Frequency	Average OD650
HEWVSYA**HPQ**FM	1/30	1.143
QKDWW**HPQ**FAWQ	1/30	1.143
LFQSWWWD**HPQ**N	1/30	1.135
YFDWLHM**HPQ**NA	1/30	1.121
MIQWWS**HPQ**FQW	1/30	1.117
KSPWFKC**HPQ**FC	1/30	1.114
WLWMYRE**HPQ**FS	1/30	1.079
DWGIIWE**HPQ**FR	1/30	1.078
PGPRYVL**HPQ**FQ	1/30	1.064
LPTRSWF**HPQ**ND	1/30	1.047
WRWLQEQ**HPQ**FR	1/30	1.028
WFEWPC**HPQ**FEV	1/30	0.925
WWFTEN**HPQ**NGM	1/30	0.899
WNPTCFY**HPQ**VQ	1/30	0.869
IHANWAR**HPQ**NM	1/30	0.827
WTFKNK**HPQ**FQS	1/30	0.754
GWPMQ**HPQ**NDMW	1/30	0.676
WKEKWWNTV**CC**T	5/30	1.155
LFINHVSEW**CC**I	1/30	1.056
LKWWNDRTV**CC**V	1/30	0.949
DGRWWQLDA**CC**I	1/30	0.914
WHMQAEWQI**CC**I	1/30	0.909
KTKFKQPNR**CC**V	1/30	0.868
YWMDTIPDWFHM	1/30	1.060
MQHPPAWLAYYH	1/30	0.956
VQFVEGWMFLEH	1/30	0.321
Media		0.041
T7 10-3b		0.041

Library screening and streptavidin-binding phage ELISAs performed as described in Krumpe et *al*., Proteomics 2006, 6, 4210-4222.
